# Direct machine parameter optimization for intensity modulated radiation therapy (IMRT) of oropharyngeal cancer – a planning study

**DOI:** 10.1120/jacmp.v10i4.3066

**Published:** 2009-09-02

**Authors:** Barbara Dobler, Oliver Koelbl, Ludwig Bogner, Fabian Pohl

**Affiliations:** ^1^ Department of Radiotherapy Regensburg University Medical Center Regensburg Germany

**Keywords:** intensity modulated radiation therapy (IMRT), direct machine parameter optimization (DMPO), direct aperture optimization (DAO), head and neck cancer, parotid sparing

## Abstract

The purpose of the study was to investigate the potential of direct machine parameter optimization (DMPO) to achieve parotid sparing without compromising target coverage in IMRT of oropharyngeal cancer as compared to fluence modulation with subsequent leaf sequencing (IM) and forward planned two‐step arc therapy (IMAT). IMRT plans were generated for 10 oropharyngeal cancer patients using DMPO and IM. The resulting dose volume histograms (DVH) were evaluated with regard to compliance with the dose volume objectives (DVO) and plan quality. DMPO met the DVO for the targets better than IM, but violated the DVO to the parotids in some cases. DMPO provided better target coverage and dose homogeneity than IM and was comparable to IMAT. Dose to the parotids (23Gy) was significantly lower than for IMAT (48Gy), but somewhat higher than for IM (20Gy). Parotid sparing with IM was, however, only achieved at the cost of target coverage and homogeneity. DMPO allows achieving parotid sparing in the treatment of oropharyngeal cancer without compromising target coverage and dose homogeneity in the target as compared to two‐step IMAT. Better overall plan quality can be delivered with less monitor units than with IM.

PACS number: 87.50.Gi

## I. INTRODUCTION

For patients undergoing radiation therapy of oropharyngeal cancer, xerostomia due to irradiation of the parotid glands is a major limitation of quality of life. Radiation dose and the salivary gland volume irradiated strongly influence the amount of saliva produced.^(1)^ Severe effects can be avoided if at least 50% of the volume of the parotid glands is kept outside the radiation field.^(2)^ The Radiation Therapy Oncology Group RTOG 0022 recommends keeping the median dose to either parotid below 30Gy.^(3)^ This goal is difficult to achieve in conventional radiation therapy of oropharyngeal cancer with a prescription dose of up to 70Gy. The parotid glands are in close proximity to or even overlapping with the planning target volume (PTV), and the primary organ at risk (OAR) to avoid is the spinal cord, which is located in a concavity of the PTV. The forward planned two‐step intensity modulated arc therapy (IMAT), as proposed by Bratengeier,^(4)^ permits good target coverage while sparing the spinal cord. Possibilities to spare the parotid glands, however, are limited in this technique.

Previous studies have reported that the use of intensity modulated radiation therapy (IMRT) spares the spinal cord and preserves the function of the parotid glands without compromising the dose to the target.^(^ ^5^ ^–^ ^16^ ^)^ Most of these studies used beamlet‐based fluence optimization with subsequent leaf sequencing. In this approach, each beam is divided into small pencil beams (beamlets). The fluences of these beamlets are optimized, leading to a modulated fluence map for each beam. The fluence map has to be converted into segments, which can be delivered by a multileaf collimator (MLC). This is done in a subsequent process, the leaf sequencing algorithm, which takes MLC constraints and the desired maximum number of segments or intensity levels into account.^(^ ^17^ ^–^ ^21^ ^)^ Another approach is to optimize the shape and weight of deliverable segments directly. MLC constraints and maximum number of segments are taken into account in the optimization process, and no subsequent leaf sequencing is required. This is referred to as direct aperture optimization (DAO) if simulated annealing is used in the optimization, and direct machine parameter optimization (DMPO) in case of a gradient descent algorithm.^(^ ^22^ ^–^ ^28^ ^)^

The aim of this study is to compare the potential of the DMPO called “Direct Step and Shoot” (DSS) versus the beamlet‐based fluence modulation with subsequent leaf sequencing (IM), both implemented in Oncentra Masterplan V.1.5 (Nucletron BV, The Netherlands) to achieve parotid sparing without compromising target coverage in IMRT of oropharyngeal cancer. Treatment plans are compared with respect to compliance with the DVO, plan quality, and monitor units required per fraction dose. Forward planned two‐step IMAT plans were used as a reference.

## II. MATERIALS AND METHODS

### A. Patients and structure definition

Ten patients with oropharyngeal cancer were included in the planning study. International guidelines for head and neck cancers were used to define the planning target volume.^(^ ^11^ ^,^ ^29^ ^–^ ^32^ ^)^ The PTV for the first series includes the ${\text{GTV}}_{P},\;{\text{GTV}}_{N}$, adjacent lymph nodes (LN), and safety margins for setup uncertainties as described by Chao et al.^(^ ^33^ ^,^ ^34^ ^)^ The PTV was separated in a cranial part $\left({\text{PTV}}_{C}\right)$, and two caudal parts, the left and right cervical lymph nodes (${\text{PTV}}_{\text{LN}}$ left, ${\text{PTV}}_{\text{LN}}$ right). The average volume of the ${\text{PTV}}_{C}$ was $(579\pm 170)\;{\text{cm}}^{3}$, the volume of ${\text{PTV}}_{\text{LN}}$ left $(109\pm 38)\;{\text{cm}}^{3}$, and of ${\text{PTV}}_{\text{LN}}$ right $(112\pm 46)\;{\text{cm}}^{3}$. The parotid glands and the spinal cord were delineated as organs at risk (OAR).

### B. Treatment planning system

The treatment planning system (TPS) Oncentra Masterplan v1.5 (Nucletron BV, Veenendal, the Netherlands) was used for all treatment planning. Oncentra Masterplan has two options for the optimization process, both products of RaySearch Laboratories AB, Sweden. In the “Intensity Modulation” (IM) option, the optimization is performed for the energy fluence of the beams and the MLC segments are created afterwards in a separate leaf sequencing process. The user can define a maximum number of segments and the sequencer will iteratively create a number of segments as close as possible and below or equal to the predefined maximum. The final dose calculation is performed based on these segments. In the direct machine parameter option “DSS”, a fluence optimization with subsequent leaf sequencing as described above is performed for a few iterations to get an initial guess for the segments. In the next step, the gradients of the objective function are calculated with respect to leaf positions and weights, and the MLC segments are optimized directly. This results in MLC segments ready for delivery without further postprocessing. Other parameters regarding the MLC segments which can be chosen by the user include the minimum number of monitor units per segment and fraction, the minimum number of adjacent open leaf pairs, and the minimum size of a segment. Both optimization methods have been dosimetrically validated for clinical use at our department.

### C. Linear accelerator

Treatment planning was performed for a Siemens Primus linear accelerator (linac) having a double focused MLC with 29 leaf pairs, with 1 cm resolution at isocenter for the 27 inner leaf pairs and 6.5 cm for the two outer leaf pairs. The photon energy used in the planning study was 6 MV.

### D. Treatment goals

In our department, a total dose of 70Gy is prescribed to the primary gross tumor volume $\left({\text{GTV}}_{P}\right)$ and the nodal gross tumor volume $\left({\text{GTV}}_{N}\right)$. The PTV for the first series up to 54Gy–56Gy in 30 fractions includes the ${\text{GTV}}_{P},\;{\text{GTV}}_{N}$, adjacent lymph nodes (LN), and safety margins. This is in good agreement with the treatment scheme proposed by RTOG 0022,^(3)^ in which the subclinical PTV is treated with 54Gy–60Gy, the gross disease PTV with 66Gy, and a boost of 4Gy–6Gy to the gross tumor PTV is optional. The planning study presented here was conducted on the first series only. This was considered sufficient for assessment of the quality of the optimization strategy.

IMRT optimization was performed on the PTV as described above, with a goal of 56Gy average dose. Since there is no option to define a DVO for the average dose in the TPS, the minimum DVO was set to 53Gy and the maximum DVO to 59Gy for the ${\text{PTV}}_{C},\;{\text{PTV}}_{\text{LN}}$ left, and ${\text{PTV}}_{\text{LN}}$ right, representing 95% and 105% of the goal dose, respectively. For the spinal cord, a maximum DVO of 35Gy was chosen; for each parotid, a maximum dose of 22Gy to 50% of the volume. To avoid hot spots in the normal tissue, a maximum DVO of 59Gy was set to the external contour. The DVO to the organs at risk were chosen relatively low with respect to the additional dose given by the boost treatment or other dose prescription schemes. For a total prescription dose of 70Gy this would correspond to a maximum dose of 44Gy to the spinal cord and 27.5Gy to no more than 50% of the parotids, which complies with the RTOG protocol 0022 for IMRT for oropharyngeal cancer.^(3)^ Since tumor control is the main goal of the treatment and parotid sparing is only desired if it does not compromise target coverage, the importance weight for the DVO to the targets was set 10 times higher than for the parotids. The DVO to the spinal cord was easily achieved; therefore, the importance weight was set equal to the parotids, even though it is a major constraint. The weight of the DVO for the external contour was chosen as high as for the target, because it was difficult to achieve especially in IM. Another reason for the choice of importance weights was that in Oncentra Masterplan, a violation of a DVO for a small volume intrinsically has a larger impact on the objective function than for larger volumes. Since the main goal of the study was to compare the two optimization strategies with respect to compliance with the DVO used for optimization, the same DVOs were used for DSS and IM and were not altered between the two techniques.

### E. Radiation technique

Two seven‐field coplanar treatment plans with beam angles of 0°, 51°, 103°, 154°, 206°, 257°, 308° were generated in Oncentra Masterplan v1.5, with a photon energy of 6 MV for each patient. Using the dose volume objectives given above, one plan was optimized with DSS, the other with IM. All optimization parameters were the same for both optimization techniques for each patient. The maximal number of segments was set to 70–100, depending on the patient geometry and complexity of the structures. The minimal open field size was set to 4 cm^2^, the minimal number of open leaf pairs to 2, and the minimal number of MU per fraction and segment to 4. Dose calculation was performed using the pencil beam algorithm with inhomogeneity correction and a dose grid resolution of 0.4 cm^3^.

The reference two‐step IMAT plans were set up with four arcs and three static beams, for a total of 11 segments as proposed by Bratengeier.^(4)^ The ${\text{PTV}}_{C}$ was irradiated with: two arcs with 181°–20° clockwise (cw) and 179°–340° counterclockwise (ccw) covering most of the ${\text{PTV}}_{C}$ without the spinal cord; three static beams with beam angles of 0°, 70°, and 290° covering the whole ${\text{PTV}}_{C}$; two additional smaller segments for beam angles 70° and 290° to avoid the use of a physical wedge; and two additional arcs with 15°–80° cw and 345°–280° ccw for dose saturation in the dorsal parts of the ${\text{PTV}}_{C}$. The ${\text{PTV}}_{\text{LN}}$ were irradiated each with one anterior‐posterior beam. Photon energy was 6 MV for all beams.

### F. Evaluation

The DSS and IM plans were evaluated and compared with respect to compliance with the DVO used for optimization and with respect to plan quality using IMAT as a reference. For evaluation of the compliance with the DVO, dose differences between the DVO and the corresponding DVH points were used. For DVO which are not fulfilled, the absolute dose difference Δ between the DVO and the DVH point for the same volume was recorded. For DVH points which fulfill the DVO, the value for Δ was set to 0. For evaluation of minimum and maximum doses, $D_{99}$ and $D_{1}$ were used respectively, in order to avoid artifacts caused by very small volumes with no clinical relevance.

Plan quality was assessed by analysis of the DVH with respect to target coverage, dose homogeneity inside the target, and dose to the OAR. The part of the ${\text{PTV}}_{C}$ and ${\text{PTV}}_{\text{LN}}$ volume receiving at least 95% of the prescription dose 56Gy ${(V}_{95})$ was used as a measure for target coverage. Dose homogeneity is described by ${H=(D}_{5}{-D}_{95})/56\text{Gy}$, where $D_{5}$ and $D_{95}$ are the isodoses encompassing 5% and 95% of the volume of interest respectively.^(35)^ The lower the value for H, the more homogeneous the plan. The volume $V_{107}$, receiving at least 107% of the prescription dose, was recorded as a measure for the size of higher dose regions. For the OAR, the median dose $D_{50}$ to each parotid and the maximum dose to the spinal cord were recorded.

As a measure of leakage dose, the number of monitor units required for a 1.8Gy fraction of a plan is compared. Statistical analysis was performed using the Student's t‐test for paired samples. Differences are considered to be significant for p‐values <0.05.

## III. RESULTS

### A. compliance to DVO

The minimum DVO to the ${\text{PTV}}_{C},\;{\text{PTV}}_{\text{LN}}$ left, and ${\text{PTV}}_{\text{LN}}$ right are met significantly closer for the plans optimized with DSS than for the plans optimized with IM. The maximum DVO was also met closer in the DSS plans for ${\text{PTV}}_{C}$ and the ${\text{PTV}}_{\text{LN}}$ left; however, no significant difference could be observed for the ${\text{PTV}}_{\text{LN}}$ right. The DVO for the parotids, on the contrary, were met significantly closer in the IM plans; no significant difference could be observed for the external contour and the spinal cord. All values and a detailed comparison of the DVO to the corresponding DVH points are given in Table 1.

**Table 1:** Dose differences between DVO and corresponding DVH points.

	DSS mean±SD	IM mean±SD	*Comparison p‐value*
${\text{PTV}}_{C}$			
$\Delta \;D_{\min}$	3.2±1.6	10.8±4.6	<0.001
$\Delta \;D_{\max}$	0.6±0.8	2.8±1.6	0.001
${\text{PTV}}_{\text{LN}}\;\text{left}$			
$\Delta \;D_{\min}$	2.3±1.2	7.0±4.4	0.002
$\Delta \;D_{\max}$	0.7±0.9	2.6±1.8	0.005
${\text{PTV}}_{\text{LN}}\;\text{right}$			
$\Delta \;D_{\min}$	2.2±1.5	8.0±4.1	0.001
$\Delta \;D_{\max}$	0.7±0.8	1.5±1.5	0.24
*Left Parotid*			
$\Delta D_{50}$	1.4±1.4	0.2±0.5	0.04
*Right Parotid*			
$\Delta D_{50}$	1.3±1.1	0.2±0.6	0.01
*Spinal Cord*			
$\Delta \;D_{\max}$	0.4±0.8	1.5±1.5	0.49
*External*			
$\Delta \;D_{\max}$	0.0±0.0	0.3±0.6	0.23

Mean values and standard deviations of the dose differences Δ between DVO and corresponding DVH points for the plans optimized with IM and DSS (given in Gy). Positive values are used for DVH points which violate the DVO. For DVH points which fulfill the DVO, the difference values are set to 0.

### B. Plan quality

Analysis of the DVH of the three plan types showed that the median dose to the parotids and the maximum dose to the spinal cord could be significantly reduced with DSS and IM as compared to IMAT, with comparable median doses to all targets. For IM, however, ${\text{PTV}}_{C}$ coverage ${(V}_{95})$ was significantly reduced, dose homogeneity inside the ${\text{PTV}}_{C}$ deteriorated, and the maximum dose to the ${\text{PTV}}_{C}$ increased. For the ${\text{PTV}}_{\text{LN}}$ and the external contour, no significant difference was observed between IM and IMAT. The plans optimized with DSS, on the contrary, showed a significantly better target coverage and dose homogeneity inside the target for both ${\text{PTV}}_{\text{LN}}$, lower maximum dose to the ${\text{PTV}}_{C}$, and lower maximum dose to the external contour as compared to IMAT.

Compared to IM, DSS optimization led to better target coverage and dose homogeneity for all targets, lower maximum dose and $V_{107}$ for the ${\text{PTV}}_{C}$ and the ${\text{PTV}}_{\text{LN}}$ left, and a median dose closer to the goal of 56Gy for the ${\text{PTV}}_{\text{LN}}$ right. Maximum dose to the spinal cord was comparable, maximum dose to the external contour was lower, and the median dose to both parotids was higher for DSS. The number of MU required increased significantly by 27% for DSS and by 69% for IM, as compared to IMAT.

**Figure 1:**
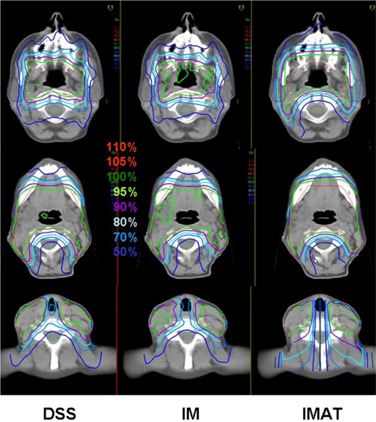
Comparison of dose distributions generated with DSS, IM and IMAT on transverse slices for a typical case.

**Figure 2:**
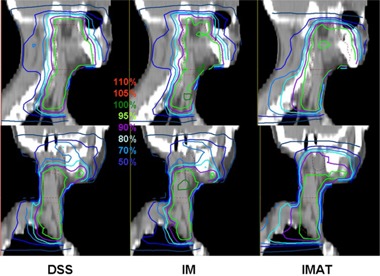
Comparison of dose distributions generated with DSS, IM and IMAT on sagittal slices for the case represented in Fig. 1.

**Figure 3:**
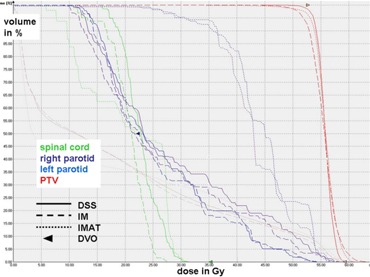
Comparison of the DVH generated with DSS, IM and IMAT for the case represented in Fig. 1.

A detailed overview over the mean values, standard deviations, and significance of improvements or deterioration of the parameters is given in Table 2. A comparison of the dose distributions generated with DSS, IM and IMAT on transverse and sagittal slices is shown for a typical case in Figs. 1 and 2. Figure 3 shows the corresponding DVH.

**Table 2:** Comparison of plan quality for IMAT, IM, and DSS.

	*IMAT*	*IM*		*DSS*		
	mean±SD	mean±SD	*vs IMAT*	mean±SD	*vs IMAT*	*vs IM*
${\text{PTV}}_{C}$						
$D_{1}$	60.5±1.0	61.8±1.6	−	59.4±1.1	+	+
$V_{95}$	91.0±4.5	68.9±18.7	−	89.6±5.4	n.s.	+
$V_{107}$	3.2±3.7	6.2±6.0	n.s.	0.8±1.2	n.s.	+
H	12.5±2.9	22.4±6.8	−	11.0±2.3	n.s.	+
${\text{PTV}}_{\text{LN}}\;\text{left}$						
$D_{1}$	60.5±0.9	61.6±1.9	n.s.	59.6±1.1	n.s.	+
$V_{95}$	83.8±5.9	73.4±16.5	n.s.	91.2±5.4	+	+
$V_{107}$	6.0±7.2	5.2±3.6	n.s.	1.0±1.6	n.s.	+
H	17.9±7.4	20.6±8.2	n.s.	10.6±2.7	+	+
${\text{PTV}}_{\text{LN}}$ *right*						
$V_{95}$	79.5±11.5	68.8±17.7	n.s.	89.5±7.4	+	+
H	15.0±3.1	18.7±5.3	n.s.	10.4±2.6	+	+
*Spinal Cord*						
$D_{1}$	37.2±3.7	33.1±3.4	+	33.7±2.5	+	n.s.
*Parotids*						
$D_{50}$ left	48.3±3.6	20.2±2.0	+	23.3±1.5	+	−
$D_{1}$ right	48.5±3.2	20.0±2.8	+	23.0±1.5	+	−
*External*						
$D_{1}$	58.9±0.8	58.5±1.2	n.s.	57.8±0.8	+	+
*#MU/1.8Gy*	744±116	1256±239	−	944±160	−	+

Mean values and standard deviations (SD) for the treatment plans resulting from the optimization with IM and DSS respectively as compared to the standard IMAT. Dose values are given in Gy, the homogeneity H in % of the goal dose of 56Gy, and volumes in % of the volume of interest. Plan quality is considered to have improved (+) or deteriorated (−) in a DVH point of interest as compared to IMAT (vs IMAT) or IM (vs IM), if p <0.05 for the students t‐test for improved or deteriorated mean values. Otherwise plan quality is considered comparable (not significant, n.s.). Parameters for which no significant changes could be observed are not listed.

## IV. DISCUSSION

The results of this study show that DSS met the DVO better than IM. Since the treatment aims at controlling the tumor, parotid sparing is desired only if it is possible to do so without putting tumor control at risk. Therefore, higher importance weights were used in the optimization for DVO to the targets than to the parotids. DSS met the DVO to the target volumes more closely than IM, leading to significantly better target coverage and dose homogeneity inside the target. In IM, the DVO to the parotids were met more closely than in DSS; they were, however, often over‐fulfilled at the cost of minimum DVO to the targets.

The plan comparison showed that better overall plan quality can be achieved with DSS optimization than with IM and IMAT. The reference IMAT plans showed good target coverage and dose homogeneity inside the target and sufficient sparing of the spinal cord. The median dose to the parotids, however, was as much as 90% of the prescription dose. With IM optimization, the median dose to the parotids could be reduced to 36% of the prescription dose. Target coverage and dose homogeneity, however, degraded at the same time significantly. The use of DSS optimization led to a reduction in median dose to the parotids to 41% of the prescription dose, with similar or even better target coverage and dose homogeneity as compared to IMAT. In all cases, maximum dose to the spinal cord and normal tissue were uncritical. Less monitor units were required per fraction with DSS than with IM, keeping the leakage dose lower. Compared to IMAT, more MU were required, which is justifiable with better overall plan quality.

**Figure 4:**
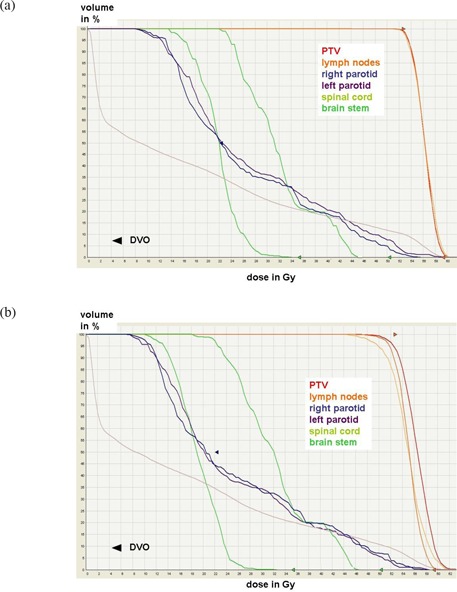
Comparison of the DVH of a typical case after IM optimization: (a) before segmentation; (b) after segmentation.

The reason for the differences in compliance to DVO and plan quality can be found in the fact that the fluence map resulting from the optimization process in IM is not deliverable without a subsequent sequencing process in which the MLC segments are created. This process decreases the number of fluence levels. The deliverable fluence map changes and leads to a dose distribution which is no longer the result of the optimization. In the cases studied here, the DVO of the PTV, LN, and the parotids are closely met in the result of the optimization with IM before MLC sequencing (Fig. 4(a)). The segmentation leads to a smeared out DHV with lower minimum and higher maximum dose (i.e. less dose homogeneity in the targets; see Fig. 4(b)). The median dose to the parotids decreases simultaneously.

**Figure 5:**
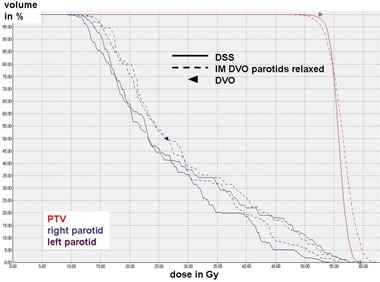
Comparison of the DVH of a typical case generated with IM with a relaxed DVO of 26Gy to the median of the parotids as compared to DSS with the standard DVO of 22Gy. Target coverage and dose homogeneity generated with IM are inferior to DSS even for relaxed DVO to the parotids.

On the other hand, in DSS the deliverable MLC shapes and weights are optimized directly (i.e. the result of the optimization is deliverable without any further processing). Figure 5 illustrates that even if the DVO to the parotids are relaxed as compared to DSS, target coverage and homogeneity are still inferior to DSS.

It might be possible to achieve better plan quality with IM using DVO other than the dose volume relations desired. Knowing that the median dose to the parotids will mostly be lower than required in the DVO, the DVO to the parotids could be relaxed, favoring higher dose to the target. To find the optimal DVO that leads to the desired result, however, is time‐consuming and requires multiple planning trials. However, with DSS, good plan quality can mostly be achieved in one or a few trials. Out of the three planning options compared in this study, DSS optimization is therefore considered optimal for treatment of oropharyngeal cancer. Keeping target coverage and dose homogeneity at the level achieved with the reference IMAT, the median dose to the parotids could be reduced to well below 30Gy, which is the recommendation of RTOG 0022,^(3)^ even for prescription schemes up to 70Gy. Previous studies have shown the advantages of DAO algorithms with simulated annealing implemented in other TPS as compared to beamlet‐based fluence optimization methods for breast cancer^(^ ^36^ ^,^ ^37^ ^)^ and prostate cancer.^(38)^ It was noted, however, that DAO might not be as powerful as beamlet‐based IMRT for head and neck cancer.^(37)^ The results of this study now show clear advantages of the gradient descent based DSS algorithm for oropharyngeal cancer, and confirm a previous report about hypopharyngeal cancer.^(39)^

## V. CONCLUSIONS

The direct machine parameter optimization DSS implemented in Oncentra Masterplan v1.5 allows parotid sparing in the treatment of oropharyngeal cancer without compromising target coverage and dose homogeneity in the target, as compared to two‐step IMAT. DSS is a major improvement compared to the beamlet‐based fluence optimization method IM, which allowed parotid sparing only at the cost of reduced dose homogeneity inside the target. Better plan quality can be delivered with less monitor units than plans optimized with IM.

## ACKNOWLEDGEMENTS

This work was partly supported by Nucletron BV, Veenendal, The Netherlands by supplying the beta version of Oncentra Masterplan v1.5 SP1. Thanks to Björn Hårdemark for information about DMPO.
